# Comprehensive analysis of the clinical significance, immune infiltration, and biological role of MARCH ligases in HCC

**DOI:** 10.3389/fimmu.2022.997265

**Published:** 2022-10-03

**Authors:** Jun Cao, Dao-yuan Tu, Jie Zhou, Guo-qing Jiang, Sheng-jie Jin, Bing-bing Su, Hao Tang, Yu-hong Tang, Ao-qing Wang, Qian Wang, Ren-jie Liu, Chi Zhang, Dou-sheng Bai

**Affiliations:** Department of Hepatobiliary Surgery, Clinical Medical College, Yangzhou University, Yangzhou, China

**Keywords:** MARCH ligases, tumor immune microenvironment, prognostic signature, bioinformatics, hepatocellular carcinoma

## Abstract

The membrane‐associated RING‐CH (MARCH) family, a member of the E3 ubiquitin ligases, has been confirmed by a growing number of studies to be associated with immune function and has been highlighted as a potential immunotherapy target. In our research, hepatocellular carcinoma (HCC) patients were divided into C1 and C2 MARCH ligase-related patterns by the non-negative matrix factorization (NMF) algorithm. Multiple analyses revealed that the MARCH ligase-related cluster was related to prognosis, clinicopathological characteristics, and the tumor immune microenvironment (TIME). Next, the signature (risk score) of the MARCH prognosis was constructed, including eight genes associated with the MARCH ligase (*CYP2C9*, *G6PD*, *SLC1A5*, *SPP1*, *ANXA10*, *CDC20*, *PON1*, and *FTCD*). The risk score showed accuracy and stability. We found that the correlations between risk score and TIME, tumor mutation burden (TMB), prognosis, and clinicopathological characteristics were significant. Additionally, the risk score also had important guiding significance for HCC treatment, including chemotherapy, immunotherapy, and transarterial chemoembolization (TACE).

## Introduction

The global mortality and morbidity rates of hepatocellular carcinoma (HCC) are seven and three, respectively, which seriously threaten human health and life (1). With poor prognosis and complicated early diagnosis owing to the lack of reliable biomarkers, despite significant improvements in anticancer therapies over the past decades, the overall survival (OS) of HCC patients is still poor (2). Chemotherapy, immunotherapy, and transarterial chemoembolization (TACE) are the preferred treatments in patients with recurrent or end-stage HCC. However, the median OS probability usually does not improve much owing to the absence of reliable biomarkers of therapeutic sensitivity (3).

Ubiquitination is a highly versatile and conserved posttranslational modification that participates in the localization and degradation of many cytosolic and membrane proteins (4) and plays a significant role in cancer tumorigenesis and progression (5, 6). It requires the sequential action of three enzymes, E1, E2, and E3 ubiquitin ligase, and the E3 ubiquitin ligase, which can determine the specificity of protein substrates, is considered a potential diagnostic and therapeutic target for cancer (7). The membrane‐associated RING‐CH (MARCH) ligases, as one of the E3 ubiquitin ligases, control the function of important immunoreceptors, including histocompatibility complex class (MHC) molecules and the costimulatory molecule CD86. In fact, numerous studies have demonstrated the important role of MHC in the anti-PD-1 treatment of tumors. MHC-I expression is well known to facilitate immune evasion; MHC-II expression is correlated with the response to PD-1 blockade treatment (8). In addition, MARCH ligases are closely related to tumor invasion and metastasis. Downregulation of MARCH 1 decreases phosphorylated p38 MAPK (p-p38 MAPK) and Stat3 (p-Stat3) and inhibits HCC cell viability (9). MARCH 6 upregulation of ATF2 promotes HCC development (10). Downregulation of MARCH 8 expression in gastric cancer cells inhibited cell growth (11). These results suggest that MARCH ligases may play an important role in the regulation of the tumor immune microenvironment (TIME). Therefore, a comprehensive analysis of the clinical significance, immune infiltration, and biological role of MARCH ligases can provide new ideas for the diagnosis and treatment of HCC.

To counter the problems mentioned above, a cluster of 367 HCC patients from the TCGA database was formed according to the expression of MARCH ligases. By multiomics analysis, the differences between MARCH ligase-related patterns were analyzed, including clinical relevance, survival analysis, and TIME. We constructed the prognostic signature (risk score), which was proven to be an independent prognostic marker. The risk score can predict the OS of HCC patients. Furthermore, we have proven the significant correlation between risk score and somatic mutation, TIME, and the efficacy of immunotherapy, chemotherapy, and TACE in HCC patients.

## Materials and methods

### Tissue samples and related clinical data

Twenty patients with primary HCC were diagnosed at the Department of Hepatobiliary Pancreatic Surgery, Northern Jiangsu People’s Hospital and received surgical treatment. HCC tissues and paired adjacent tissues (*n* = 20 for both) were obtained from the patients in our hospital. The present study was performed in accordance with the principles outlined in the Declaration of Helsinki. Written informed consent was obtained from the individuals who provided the samples.

### Acquisition and process of original data

From the TCGA database (https://portal.gdc.cancer.gov/), we obtained the transcriptional RNA sequencing, somatic mutation, and clinical information of the TCGA-HCC cohort. Our transcript RNA sequencing panel included 374 HCC tumor tissues. These data were compiled as fragments per kilobase of transcript per million mapped reads (FPKM). When an individual gene symbol included more than one Ensembl ID, the calculation method annotated the gene expression in an average. A total of 367 patients were included in the training set after excluding the samples without complete information on OS. In this study, 350 TCGA samples had somatic mutation information. The 221 HCC samples with clinical information and RNA-seq data were provided by the GEO (https://www.ncbi.nlm.nih.gov/gds) to be used as the external test set. The copy number mutation landscape of 11 MARCH ligase genes was plotted, which are the mammalian homologs of K3 and K5 identified by bioinformatics studies (9), employing the R package “Rcircos” in human chromosomes. We used the GSE104580 (10) and GSE109211 (12) chips in the study to analyze TACE and sorafenib sensitivity.

### Non-negative matrix factorization clustering

To explore the distinct MARCH ligase-related patterns, we applied non-negative matrix factorization (NMF) through the R package “NMF.” The original matrix is subdivided into two non-negative matrices based on the NMF algorithm (13) to identify the potential characteristics of the gene expression profile. To obtain consistent clustering, the algorithm repeats the deposition and aggregates the result. *K* = 2 is the best cluster value, according to the cophenetic coefficient, sample size, and contour. Through the R package “prcomp,” the principal component analysis (PCA) scoring system was constructed by all the MARCH ligase-related genes that were selected.

### Gene set enrichment analysis

Gene set enrichment analysis (GSEA) is an algorithm that is non-parametric and unsupervised that can convert the separated gene expression matrix into a particular gene set as a characteristic expression matrix. The algorithm is performed by the R packages “clusterProfiler,” “enrichplot,” and “DOSE.” We adopted the package “limma” to analyze the significant differences after transformation in the expression matrix. Through the R package “GSVA”, we investigated the Kyoto Encyclopedia of Genes and Genomes (KEGG) pathway by gene set variation analysis (GSVA). “h.all.v7.5.1.symbols.gmt” was obtained from the GSEA database.

### Evaluation of the TIME

The tumor immune dysfunction and exclusion (TIDE) score is a computational approach for predicting the immune escape of tumor cells; thus, a higher TIDE score suggests a lower response rate to immune checkpoint inhibitor (ICI) treatment. To model the mechanisms of distinct tumor immune evasion, the superior algorithm (14) was used to evaluate the TIDE.

To calculate the fractions of immune cell types in each sample, the MCP-counter method was adopted to determine the characteristics of the TIME. Additionally, through the Wilcoxon rank-sum test, we evaluated the relevance between immune cell abundance and risk score. We used the single-sample GSEA (ssGSEA) to study the immune status.

### Functional enrichment analysis of differentially expressed genes

To identify differentially expressed genes (DEGs) between two different phenotypes, we adopted the package “limma” to assess the differences in gene expression after NMF clustering by *p*-values (*p*< 0.001) and *t*-statistics. Then, to analyze DEGs between MARCH ligase-related patterns, we used the enrichment of the KEGG and gene ontology (GO) pathways by the Metascape web-based platform (15).

### Establishment and validation of the prognostic signature

The least absolute shrinkage and selection operator (LASSO) was performed by the package “glmnet” based on the DEGs that were prognosis-related in the model of univariate Cox regression; the important prognostic genes included eight DEGs (*CYP2C9*, *G6PD*, *SLC1A5*, *SPP1*, *ANXA10*, *CDC20*, *PON1*, and *FTCD*) that were identified by minimum criteria. Finally, we obtained the risk score formula:


f(x)=∑(exp Gene×coeffient Genei)

The TCGA-HCC cohort was divided into low- and high-risk groups through the R package “surv_cutpoint” after calculating the optimal cutoff of risk score. Through the receiver operating characteristic (ROC) curve (package of “timeROC”) and Kaplan–Meier analysis (package of “survival”), we evaluated the predictive reliability of the prognostic models. The area under the curve (AUC) was applied to quantify the ROC curve. To validate the signature in the GEO cohort, we used the same analysis methods, risk score formula of calculation, and cutoff value.

### Single-cell RNA-seq analysis

The single-cell RNA-seq data are available in the GEO database, reference chip GSE146115 (16). The chip of genes in each cell is in the range of 50 to 9,000, the percentage of red blood cells is less than 3%, and the total gene expression copy numbers are less than 300,000. We selected 1,500 genes with the largest variances and labeled them in red, and we tagged the names of the first 10 genes that were highly variable at the same time.

First, PCA was utilized for dimension reduction based on the highly variable genes, and the resolution was set to 0.5. In total, we obtained 12 clusters. Cell clusters were visualized using UMAP algorithms, and the first 10 genes that showed significant differences in each cluster were selected and mapped. Subsequently, identifying the top differentially expressed genes for each cluster was performed using the FindAllMarkers function. In addition, the expression pattern of each marker gene among clusters was visualized by applying the “DotPlot” function in Seurat. Afterward, the SingleR package (version 1.10.0) was employed for marker-based cell-type annotation.

### Efficacy evaluation of chemotherapy and targeted drugs

To calculate the half-maximal inhibitory concentration (IC_50_), we adopted the ridge regression algorithm, and through 10-fold cross-validation (17), we obtained satisfactory prediction veracity. The process was calculated through the package “pRRophetic” in R.

### Quantitative real-time PCR

According to the manufacturer’s protocol, total RNA was extracted by TRIzol reagent (Invitrogen Carlsbad, CA, USA) from 20 paired HCC and paracancerous tissues. Then, total RNA from each sample was reverse-transcribed to cDNA using the PrimeScript™ RT reagent Kit (Takara Bio Inc., Japan). Real-time PCR was conducted using the SYBR-Green PCR kit (Takara, Osaka, Japan) in a Rotor-Gene 3000 machine (Corbett Life Science, Sydney, Australia). The specific primers used are listed in **Supplementary Table 1**.

### Statistical analysis

The R software (version 4.0.5) was used to perform all statistical analyses in this study. To confirm a significant difference between the two groups, we used a paired-samples *t*-test or Wilcoxon rank-sum test. To check if there was a significant difference in more than two groups, the Kruskal–Wallis test was used. The relevance coefficients among the expression of immune checkpoint genes, tumor mutation burden (TMB), and risk score were calculated through Spearman’s correlation analysis. To indicate the gene mutation frequency, we built waterfall plots by the package “maftools” and set a standard of significant difference as *p*-value<0.05.

## Results

### The landscape of genetic variation of MARCH ligase regulators in HCC

**Figure 1:**
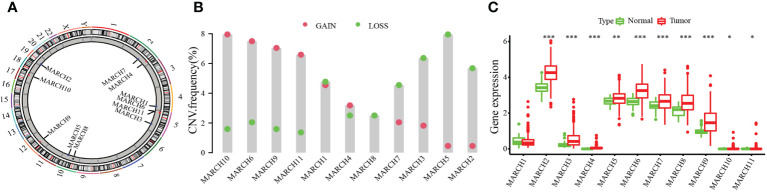
The genetic alterations of the membrane‐associated RING‐CH (MARCH) ligase landscape in hepatocellular carcinoma (HCC). **(A)** The CNV alteration location of MARCH ligase on chromosomes. **(B)** The CNV mutation frequency of 11 MARCH ligases was prevalent. The column represents the alteration frequency. The deletion frequency, blue dot; the amplification frequency, pink dot. **(C)** Differences in the gene expression levels of 11 MARCH ligases between normal and tumor samples. The asterisks represent statistical *p*-value (*p< 0.05; **p< 0.01; ***p< 0.001).

We researched the roles of 11 MARCH ligases (MARCH 1–11) in HCC. The analysis of 11 MARCH ligases showed that copy number variation (CNV) mutations were prevalent. MARCH 4, MARCH 6, MARCH 9, MARCH 10, and MARCH 11 revealed widespread CNV amplification. **Figure 1A** shows the locations of CNV alterations of 11 MARCH ligase genes on chromosomes. In contrast, MARCH 1, MARCH 2, MARCH 3, MARCH 5, MARCH 7, and MARCH 8 had prevalent CNV deletions (**Figure 1B**). Further analysis demonstrated that MARCH 2–11 were significantly upregulated in HCC samples (**Figure 1C**, **Figure Supplement 2**). The above results suggested that MARCH ligase may play a significant role in modulating the progression of HCC.

### NMF clustering of MARCH ligase-related patterns

**Figure 2:**
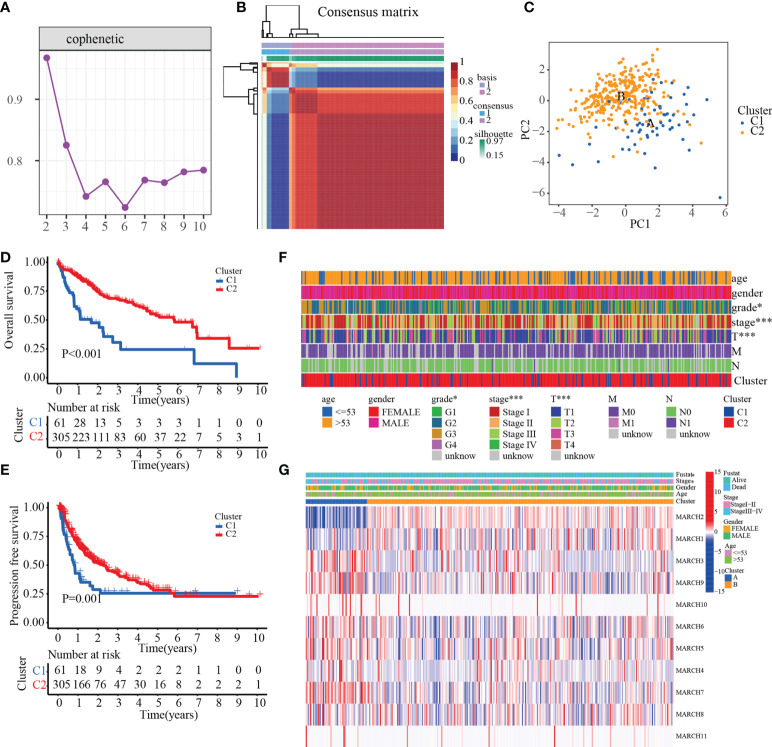
The prognostic value of C1 and C2. **(A)** Non-negative matrix factorization clustering of necroptosis-related patterns in the TCGA-HCC cohort. **(B)** Cophenetic coefficients. Expression profile of prognosis-related MARCH ligase gene: **(C)** PCA and **(D, E)** Kaplan–Meier analysis of OS and PFS. **(F)** Clinical relevance of MARCH ligase-related patterns. **(G)** Transcription profile heatmap of 11 prognosis-related MARCH ligase genes in C1 and C2 (*p< 0.05; ***p< 0.001).

According to 11 MARCH ligases in the univariate Cox regression model, NMF clustering was used on the TCGA-HCC cohort. *K* = 2 was the best clustering result based on cophenetic coefficients (**Figures 2A, B**). The PCA results illustrated the differences between the C1 and C2 patterns at the transcriptional level of the MARCH ligases (**Figure 2C**). Compared with the C1 pattern, the Kaplan–Meier analysis suggested that the OS or progression-free survival (PFS) of the C2 pattern was significantly longer (**Figure 2D**, *p*< 0.001; **Figure 2E**, *p* = 0.001). Lastly, we adopted the chi-square test to reveal the difference between the C1 and C2 patterns in the clinicopathological characteristics. As the figure demonstrates, the pathologic stage, T stage, and histologic grade distribution were noticeably different in the C1 and C2 patterns (**Figure 2F**). The transcription profile heatmap of 11 MARCH ligase genes was significantly different in the C1 and C2 patterns (**Figure 2G**).

### TIME of MARCH ligase-related patterns

**Figure 3:**
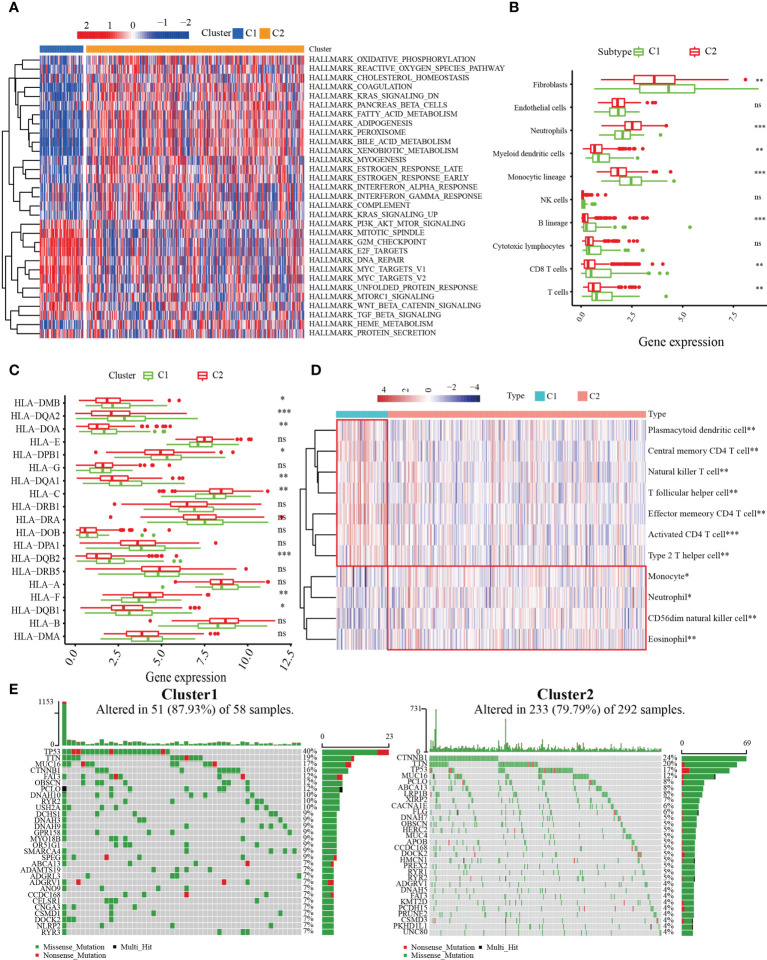
Correlation between MARCH ligase-related patterns and the tumor immune microenvironment (TIME). **(A)** Heatmap of GSVA results. **(B)** The difference in TIME composition. **(C)** Differential expression levels of HLA-related genes between the C1 (green) and C2 (red) groups (ns = not significant, *p< 0.05, **p< 0.01, ***p< 0.001). **(D)** Heatmap showing the relative abundances of 11 immune-infiltrating cell subpopulations. **(E)** The 30 genes with the highest mutation rates of C1 and C2 in waterfall plots.

To explore the biological molecular changes between the C1 and C2 patterns, we performed GSVA to explore the biological processes among these distinct RNA processing patterns (**Figure 3A**). The results showed that C1 had a significantly higher concentration in the reactive oxygen species pathway, oxidative phosphorylation, k-ras signaling, metabolism-related, and INF-α/γ response. However, in the PI3K–AKT–MTOR signaling, G2/M checkpoint, DNA repair, MYC target, and unfolded protein response pathways, C2 had a higher concentration. The results demonstrated that MARCH ligase may affect the prognosis of HCC patients by regulating the TIME. Therefore, we quantified the composition of the TIME using MCP-counter analysis to study the discrepancy in the immune-related characteristics between C1 and C2. We found that the infiltration rates of fibroblasts, myeloid dendritic cells, monocytic lineage, T cells, and B cells in C1 were higher than those in C2 (**Figure 3B**). Because the MARCH ligases could reduce the expression of MHC molecules, we researched the expression of MHC molecule-related genes between the C1 and C2 patterns. The results showed that the C1 cluster expression levels were higher than the C2 cluster in most major MHC molecules (**Figure 3C**). Additionally, to compare and visualize the correlative richness of 11 subpopulations of immune-infiltrating cells between the C1 cluster and C2 cluster, we established a heatmap with ssGSEA. We discovered that T follicular helper cells, CD4^+^ T cells, and NK cells were enriched in the C1 cluster (**Figure 3D**). As the waterfall plots showed, the 10 genes with the highest mutation rates in C1 were *TP53* (40%), *TTN* (19%), *MUC16* (17%), *CTNNBI* (16%), *FAT3* (12%), *OBSCN* (12%), *PCLO* (12%), *DNAH10* (12%), *RYR2* (10%), and *USH2A* (10%). In contrast, the 10 genes with the highest mutation rates in C2 were *CTNNB1* (24%), *TTN* (20%), *TP53* (17%), *MUC16* (12%), *PCLO* (8%), *ABCA13* (8%), *LRP1B* (7%), *XIRP2* (6%), *CACNA1E* (6%), and *FLG* (5%) (**Figure 3E**). In summary, we confirmed that C1 has more immune-related mutations.

To further construct the MARCH ligase-related prognostic score, we researched the DEGs between the C1 and C2 patterns. A total of 234 differentially expressed genes were screened, of which 24 genes were downregulated and 210 genes were upregulated (**Figure Supplement 3A**). We applied functional enrichment analyses of DEGs among the C1 and C2 patterns to study the differences in molecular GO, indicating that DEGs primarily participated in metabolic processes, biosynthetic processes, complement and coagulation cascades, nuclear receptor meta-pathways, and complement cascades (**Figure Supplement 3B**).

### Establishment of the MARCH ligase signature in the TCGA-HCC cohort

**Figure 4:**
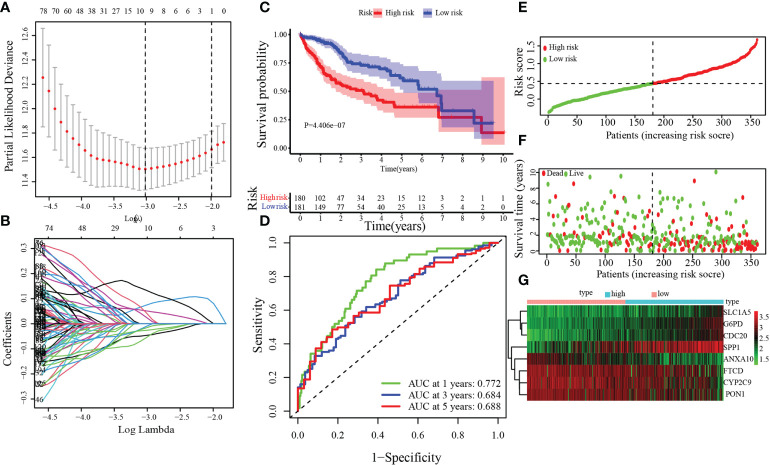
Establishment of the MARCH ligase signature according to the training set. **(A, B)** LASSO Cox regression analysis. **(C)** Kaplan–Meier analysis between risk score-defined groups. **(D)** Time-dependent ROC curve of risk score. **(E)** Risk score distribution. **(F)** Survival status heatmap. **(G)** The expression profile heatmap of eight genes.

The univariate Cox regression analysis model was obtained based on previous research with DEGs among C1 and C2. Next, by analyzing the LASSO Cox regression, we processed the univariate Cox regression model to obtain the coefficient, and eight genes, *CYP2C9*, *G6PD*, *SLC1A5*, *SPP1*, *ANXA10*, *CDC20*, *PON1*, and *FTCD*, were chosen based on the minimum standard (**Figures 4A, B**). The quantitative indicator was obtained: risk score = (0.08152 × *G6PD* expression) + (−0.02512 × *CYP2C29* expression) + (0.00272 × *SLC1A5* expression) + (0.05107 × *SPP1* expression) + (−0.04573 × *ANXA10* expression) + (0.12660 × *CDC20* expression) + (−0.00739 × *PON1* expression) + (−0.03437 × *FTCD* expression). Next, we calculated the risk score according to the above formula for each patient. Based on the optimal cutoff value (cut point = 0.4380) for the risk score, the patients were divided into a low-risk group (*n* = 181) and a high-risk group (*n* = 180) through Kaplan–Meier analysis. Compared with the high-risk group, the OS in the low-risk group was obviously longer (**Figure 4C**, *p* = 4 × 10^−7^). In addition, to predict the OS survival rate of HCC patients, we used ROC curves to evaluate the risk score veracity. **Figure 4D** demonstrates that 0.772, 0.684, and 0.688 were the 1-, 3-, and 5-year OS survival rates of AUC, respectively. In the high-risk group, the ratio of deaths was elevated with risk score (**Figures 4E, F**). With the risk processes, the expression of *G6PD*, *SLC1A5*, *SPP1*, and *CDC20* was increased, and *CYP2C9*, *ANXA10*, *PON1*, and *FTCD* expression was negatively correlated with the risk score (**Figure 4G**).

### MARCH ligase signature validation in the GEO-HCC cohort

**Figure 5:**
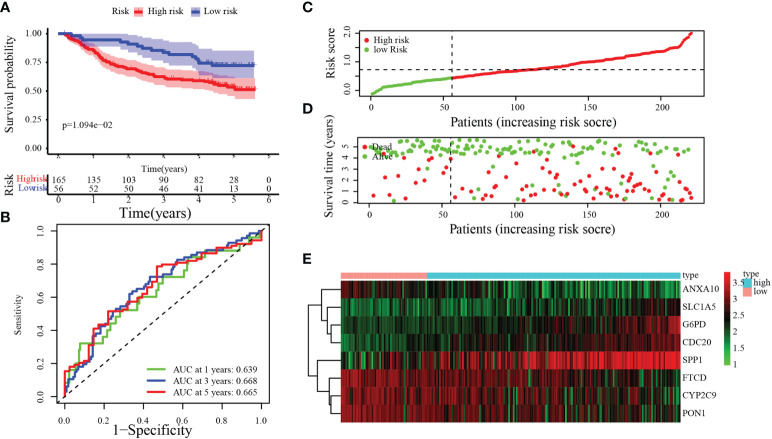
Validation of the MARCH ligase signature based on the test set. **(A)** Kaplan–Meier analysis between the risk score-defined groups. **(B)** Time-dependent ROC curve of risk score. **(C)** Risk score distribution. **(D)** Survival status heatmap. **(E)** The expression profile heatmap of eight genes.

To verify the accuracy and stability of our findings, the risk score model was further evaluated using the GEO dataset, which included 221 HCC patients. According to the same cutoff value (cut point = 0.4380), groups were divided into the training set [low-risk group (*n* = 56) and high-risk group (*n* = 165)]. By Kaplan–Meier analysis, we found that the OS of patients in the high-risk group was significantly poorer than that in the low-risk group (**Figure 5A**, *p* = 1.094 × 10^−2^). **Figure 5B** demonstrates that 0.639, 0.668, and 0.665 were the 1-, 3-, and 5-year OS survival rates of AUC, respectively. The trends of the training sets and test sets were similar in the risk score distribution, the status of survival, and the heatmaps of the expression profile (**Figures 5C–E**).

### Clinical relevance of the MARCH ligase signature

**Figure 6:**
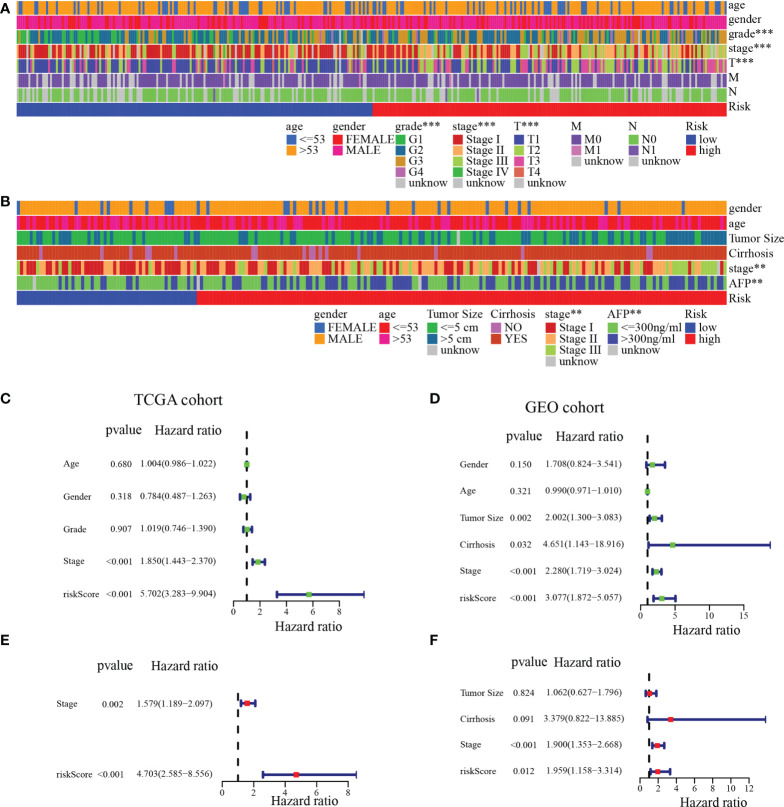
Independent prognostic analysis of risk score. **(A)** Clinical relevance of the high-risk and low-risk groups in the TCGA cohort. **(B)** Clinical relevance of the high-risk and low-risk groups in the GEO cohort. Univariate **(C)** and multivariate **(E)** Cox regression analyses of risk score and clinicopathological parameters in the TCGA cohort. Univariate **(D)** and multivariate **(F)** Cox regression analyses of risk score and clinicopathological parameters in the GEO cohort. **p< 0.01; ***p< 0.001.

To further analyze the clinical significance of the MARCH ligase signature, we studied the relevance of the risk score and clinicopathological characteristics. In the TCGA cohort, there was a significant difference between the low- and high-risk groups in terms of histologic grade, pathologic stage, and T stage (**Figure 6A**). In the GEO cohort, there was a significant difference between the low- and high-risk groups in the pathologic stage and the serum alpha-fetoprotein (AFP) content, which is a serum marker of HCC (**Figure 6B**). In summary, the tendency for poor prognosis results and advanced pathological characteristics was concentrated in the high-risk groups. Then, to study whether risk score could be an independent prognostic indicator of TCGA-HCC and GEO-HCC patients, univariate and multivariate Cox regression analyses were used. Through univariate Cox regression analysis, we found that risk score and pathological stage were also hazard factors in the TCGA cohort (**Figure 6C**). In the GEO cohort, univariate Cox regression analysis indicated that risk score was a hazard factor similar to tumor size, pathologic stage, and cirrhosis (**Figure 6D**). Next, through the analysis of multivariate Cox regression, we suggested that risk score was an excellent independent prognostic indicator both in the TCGA-HCC cohort [hazard ratio (HR) = 4.703 (2.585–8.556), *p*< 0.001, **Figure 6E**] and the GEO-HCC cohort [HR = 1.959 (1.158–3.314), *p* = 0.012, **Figure 6F**].

### Comparison of the MARCH ligase signature model and other models

**Figure 7:**
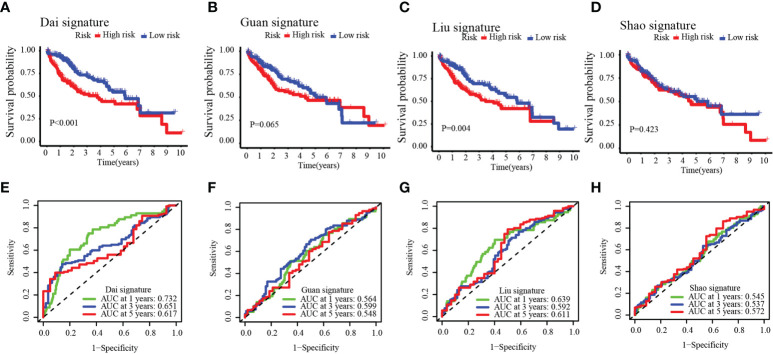
Four other prognostic models in the TCGA-HCC cohort **(A–D)** and time-dependent ROC curves **(E–H)**.

To further verify the accuracy of our model, we compared our established model with four other predictive models of precision in determining HCC prognosis: the Dai signature prognostic model (18), the Guan signature prognostic model (19), the Liu signature prognostic model (20), and the Shao signature prognostic model (21). With a similar method that established our eight-gene-based model (described above), the relevant gene risk score in the dataset of the four models was calculated for TCGA-HCC. To predict the OS of the low- and high-risk groups, the Dai signature predictive model (log-rank *p*< 0.001) and the Liu signature predictive model (log-rank *p* = 0.004) showed significant differences; the Guan signature predictive model and the Shao signature predictive model showed no significant differences (**Figures 7A–D**). Except for the Dai signature, the predictive model for the 1-year survival rate had minutely higher AUC values than our model. The 1-year survival rate of the AUC value in the Liu signature predictive model was the same as that in our model. The AUC value of our model was significantly higher than that of the other models for the 1-, 3-, and 5-year survival rates (**Figures 7E–H**). In predicting the survival of HCC patients, these results suggested that our model possessed better accuracy.

### Correlation between the TIME and MARCH ligase signature

**Figure 8:**
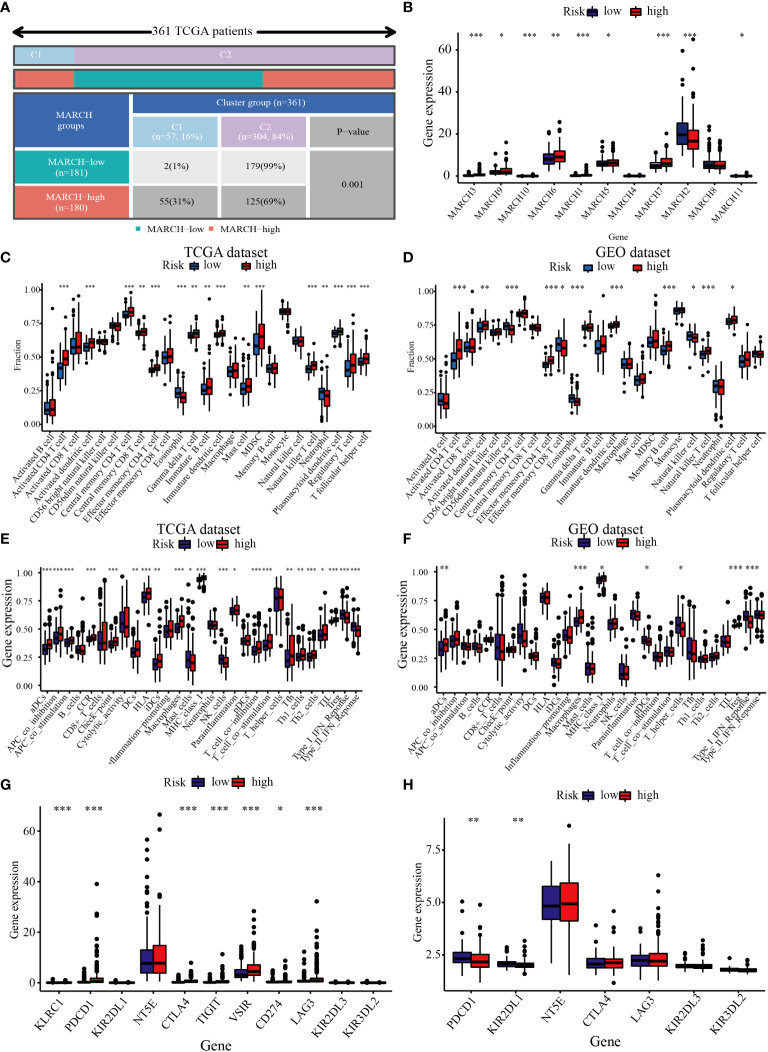
Correlation between the TIME and MARCH ligase signature. **(A)** The distribution of high-risk groups and low-risk groups in the C1 group and C2 group. **(B)** Expression of the MARCH 1–11 genes in the low-risk group and high-risk group. The ssGSEA results of different risk groups in the TCGA cohort **(C, E)** and the GEO cohort **(D, F)**. The 25 immune cell scores **(C, D)** and 13 immune-related functions **(E, F)** are shown in boxplots. **(G, H)** The relationship between risk groups and immune checkpoint expression levels. Adjusted *p* is shown as follows: ns, not significant; *p< 0.05; **p< 0.01; ***p< 0.001.

Next, we analyzed the relationship between the TIME and the MARCH ligase signature. In the 361 TCGA cohort, we analyzed the distribution in the C1 group and the C2 group of low- and high-risk groups. The analysis showed that the low-risk groups were almost all concentrated in C2, and the C1 group consisted of the high-risk groups (**Figure 8A**). In addition, MARCH 1–11 gene expression was also analyzed in the low- and high-risk groups. The results showed that compared with the low-risk group, most of the MARCH genes (MARCH 1, 3, 5, 6, 7, 9, 10, 11) were more highly expressed in the high-risk group (**Figure 8B**). The above results indicated that risk score could reflect MARCH ligase-related patterns. The cellular underpinnings of immunotherapy are CD4^+^ T cells, CD8^+^ T cells, NK cells, and other immune cells (22). Therefore, the study of immune infiltrates in the TIME is the key to improving the therapeutic response rate of HCC. Through ssGSEA, we calculated the subpopulations of different immune cells and related functional enrichment scores to study the relationship between immune status and risk score. According to the results of the analysis, CD4^+^ T cells, CD8^+^ T cells, activated dendritic cells, immature dendritic cells, and NK cells had higher levels of infiltration in patients with high-risk scores in both datasets (Wilcoxon test, *p*< 0.001) (**Figures 8C, D**). In the TCGA-HCC and GEO-HCC datasets, ssGSEA indicated that the functions of most immune-related genes were mainly enriched in the high-risk group (**Figures 8E, F**). Additionally, we further explored the immune checkpoint molecule expression among the low- and high-risk groups in both cohorts. The results indicated that the difference in PDCD1 expression was statistically significant in the low- and high-risk groups. In the TCGA dataset, there were statistically significant differences in CTLA4 expression between the low-risk group and the high-risk group (**Figures 8G, H**).

### HCC single-cell subpopulations

**Figure 9:**
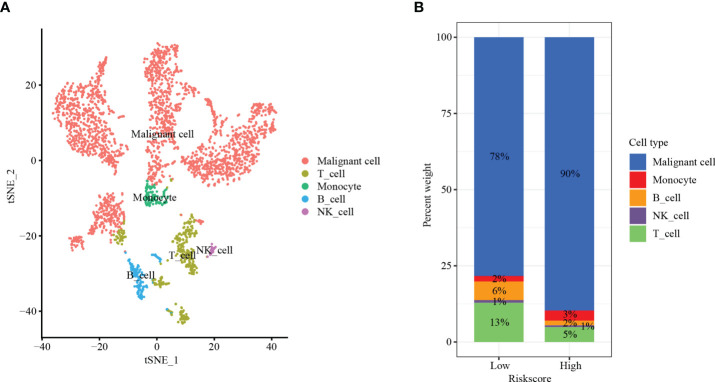
Single-cell sequencing. **(A)** t-SNE plot of 3,200 cells from 12 HCC patients showing eight major cell types. **(B)** The distribution of HCC cells, T lymphocytes, monocyte cells, B lymphocytes, and NK cells in the low- and high-risk groups.

To further verify the above results, single-cell sequencing was conducted. After stringent quality control, we obtained 3,200 single cells. We found that the cells were organized into 12 clusters after dimension reduction through PCA (**Figure Supplement 4**). The expression of the first 10 genes in each cluster was significantly higher than that in the other clusters. We categorized these clusters into HCC cells, T lymphocytes, monocyte cells, B lymphocytes, and NK cells (**Figure 9A**). Interestingly, the low-risk group had higher T lymphocytes (13% *vs*. 5%) and B lymphocytes (6% *vs*. 2%) than the high-risk group. Moreover, the proportion of HCC cells in the high-risk group was significantly higher than that in the low-risk group (90% *vs*. 78%) (**Figure 9B**). Our results indicated that risk score can correctly reflect the TIME and may have important clinical value in predicting the efficacy of tumor ICIs.

### Relationship between the MARCH ligase signature and somatic mutations

**Figure 10:**
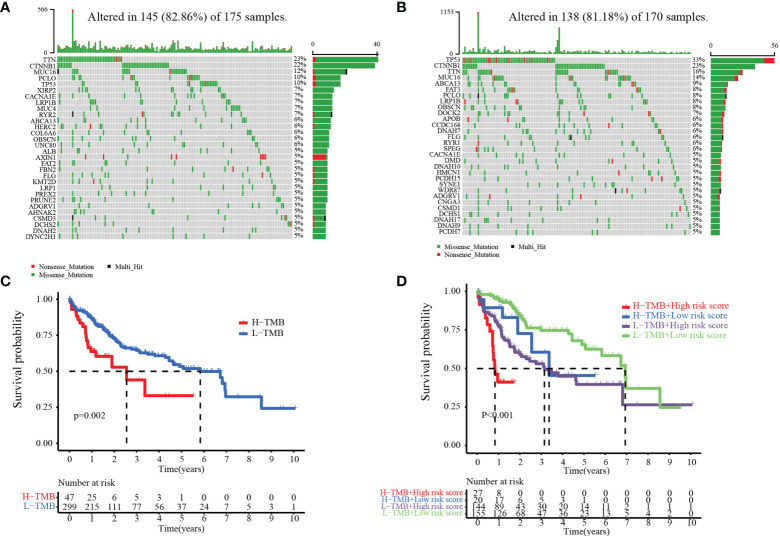
Relationship between the MARCH ligase signature and somatic mutation. Waterfall plots of 30 genes with the highest mutation rates in the high-risk group **(A)** and the low-risk group **(B)**. **(C)** Kaplan–Meier analysis of TMB in HCC patients. **(D)** Kaplan–Meier analysis of the correlation between risk score and TMB.

The accumulation of mutations often leads to the development of tumors and TIME remodeling (23). Therefore, we studied the distinction of somatic mutations between the low- and high-risk groups. The top 5 genes with the highest mutation frequencies were *TIN* (23%), *CTNNB1* (22%), *MUC16* (12%), *PCLO* (10%), and *TP5316* (10%) in the high-risk group (**Figure 10A**) and *TP53* (33%), *CTNNB1* (23%), *TIN* (16%), *MUC16* (14%), and *ABCAB* (9%) in the low-risk group (**Figure 10B**). The results showed that the low-risk group had more immune-related mutations. Next, we divided patients into low- and high-TMB groups by applying the optimal TMB cutoff. The results showed that the higher TMB value of HCC patients was associated with lower OS (**Figure 10C**, *p* = 0.002). We divided TCGA patients into four groups of high-TMB + low-risk, high-TMB + high-risk, low-TMB + low-risk, and low-TMB + high-risk based on the risk score and the optimal TMB cutoff value. The low-TMB + low-risk group had a significantly higher OS than the high-TMB + high-risk group (**Figure 10D**, *p*< 0.001).

### Relationship between the MARCH ligase signature and drug sensitivity and TACE

**Figure 11:**
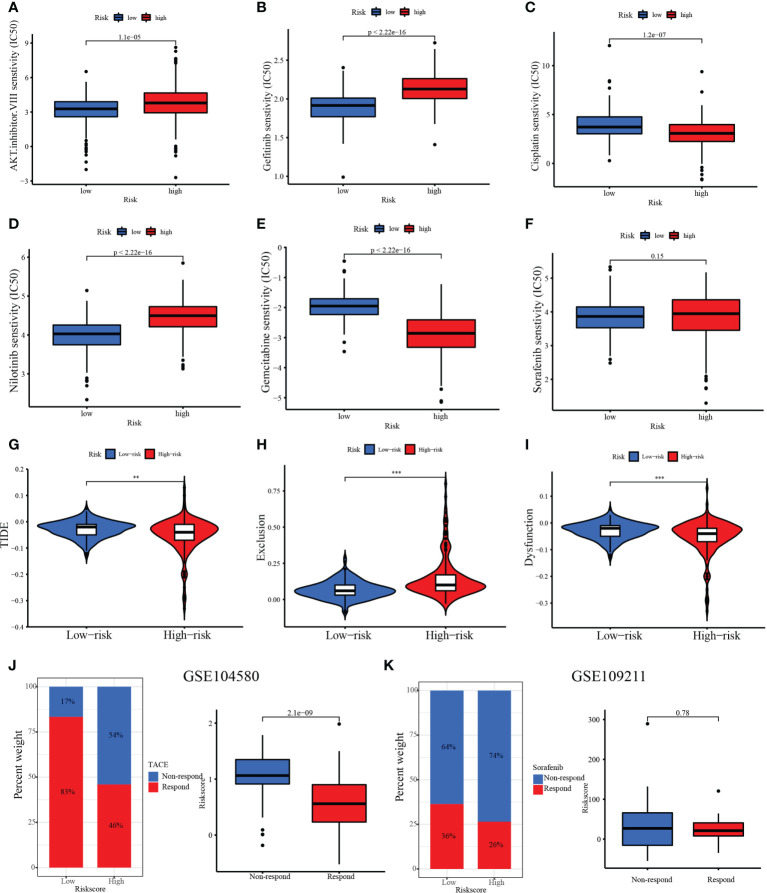
Therapeutic benefit of risk score. **(A–F)** Correlation between the MARCH ligase signature and IC_50_ values of chemotherapy and targeted drugs, including **(A)** AKT inhibitor VIII, **(B)** gefitinib, **(C)** cisplatin, **(D)** nilotinib, **(E)** gemcitabine, and **(F)** sorafenib. **(G)** The relative distribution of TIDE was compared between the low- and high-risk groups. **(H)** The relative distribution of exclusion was compared between the low- and high-risk groups. **(I)** The relative distribution of dysfunction was compared between the low- and high-risk groups. **(J)** The distribution of the transarterial chemoembolization (TACE)-responsive group versus the TACE-non-responsive group in the low- and high-risk groups and the relative distribution of risk score in the TACE-responsive group versus the TACE-non-responsive group. **(K)** The distribution of the TACE-responsive group versus the sorafenib-non-responsive group in the low- and high-risk groups and the relative distribution of risk score in the sorafenib-responsive group versus the sorafenib-non-responsive group. **p< 0.01; ***p< 0.001.

Chemotherapy and targeted drugs have demonstrated clinical benefits in the treatment of advanced HCC. Nevertheless, adverse drug reactions and resistance are still significant obstacles to the development of drug therapy (24). TIME and SNV are important factors in the therapeutic effect of HCC. Therefore, it is vital to explore a reliable predictive drug-sensitive marker to guide medication. Then, the relationships between risk score and chemotherapy, targeted drugs, and ICIs were calculated. Our results show that the IC_50_ values of the AKT inhibitor VIII, gefitinib, and nilotinib were higher in the high-risk group than in the low-risk group, while the IC_50_ values of cisplatin and gemcitabine were lower in the high-risk group than in the low-risk group. However, there was no significant difference in the IC_50_ value of sorafenib between the low- and high-risk groups (**Figures 11A–F**). Our study also confirmed that the TIDE score was decreased in the high-risk group (TIDE distribution in TCGA-HCC, **Figure 11G**, *p*< 0.01). Compared with patients in the low-risk group, patients in the high-risk group exhibited higher scores of exclusion (**Figure 11H**), whereas patients in the low-risk group expressed higher scores of dysfunction compared with the high-risk group (**Figure 11I**). In addition, the treatment response to TACE was further analyzed between the low- and high-risk groups. We observed that 17% of patients had non-response to TACE in the low-risk group, and 54% of patients had non-response to TACE in the high-risk group in the GSE104580 TACE chip (**Figure 11J**). Furthermore, the TACE-responsive group had lower scores than the TACE-non-responsive group (**Figure 11J**). The GSE109211 chip was used to study the sorafenib response rates between the low- and high-risk groups. Although the low-risk group had higher response rates than the high-risk group (36% *vs*. 26%), there was no significant difference in treatment response to sorafenib among the low- and high-risk groups (**Figure 11K**). The results showed that risk score may have important implications for the treatment of HCC, including chemotherapy, TACE, and ICIs.

### Biological function and pathway analyses

**Figure 12:**
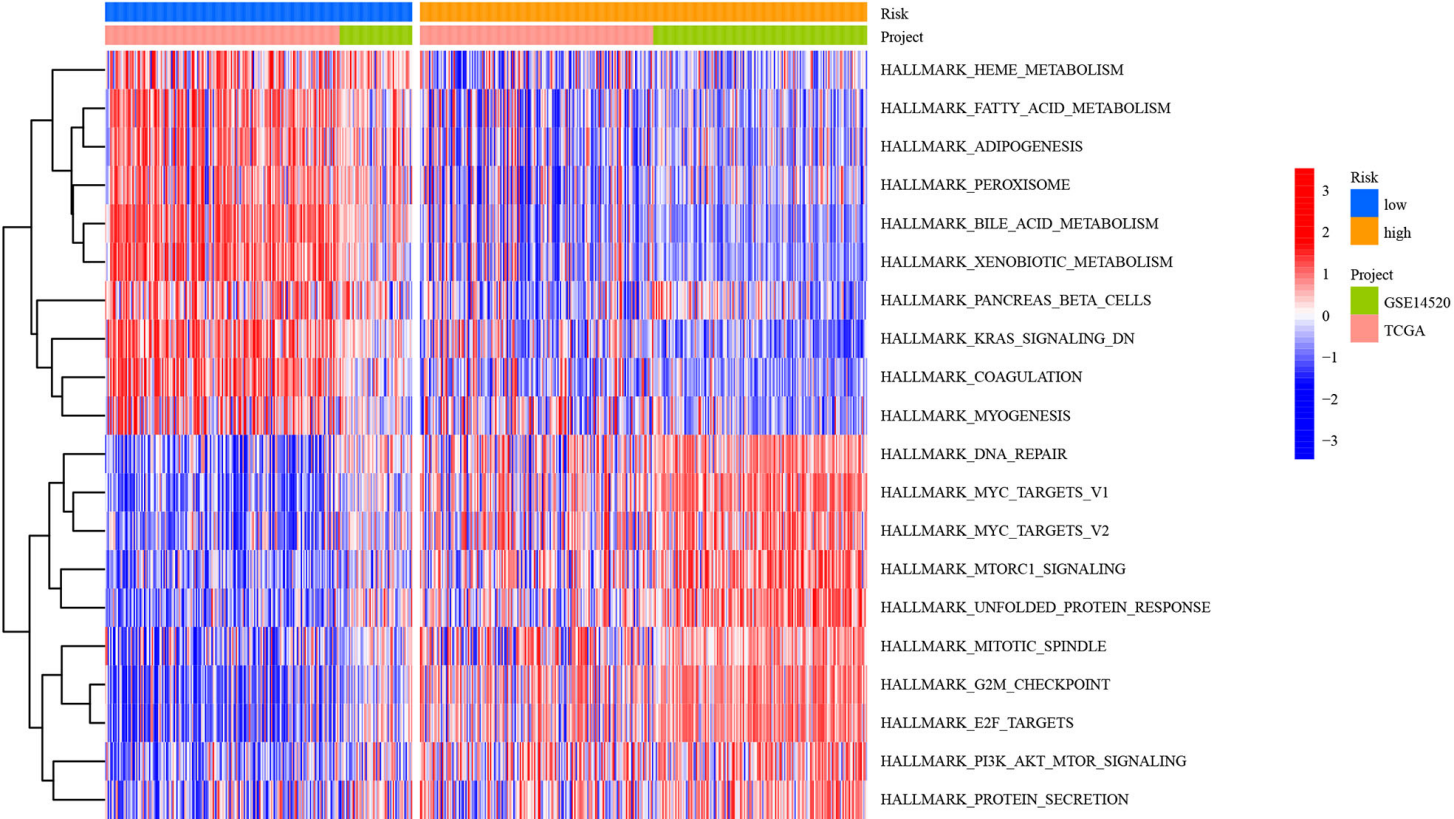
Heatmap showing GSVA scores of the hallmark gene sets for the five defined RNA processing patterns.

To explore the biological molecular changes among the low- and high-risk groups, we performed GSVA to explore the biological processes among these distinct RNA processing patterns (**Figure 12**). The low-risk groups were significantly enriched in metabolism-related processes, including heme metabolism, fatty acid metabolism, adipogenesis, peroxisome, bile acid metabolism, xenobiotic metabolism, pancreas beta cells, K-ras signaling, coagulation, and myogenesis. However, carcinogenic activation and related signaling pathways, such as DNA repair, MYC targets, mTORC1 signaling, unfolded protein response, mitotic spindle, G2/M checkpoint, E2F targets, and protein secretion, were significantly enriched in the high-risk group. As mentioned above, the results showed the obvious distinction between the low- and high-risk groups in biological function.

### Quantitative real-time polymerase chain reaction

**Figure 13:**
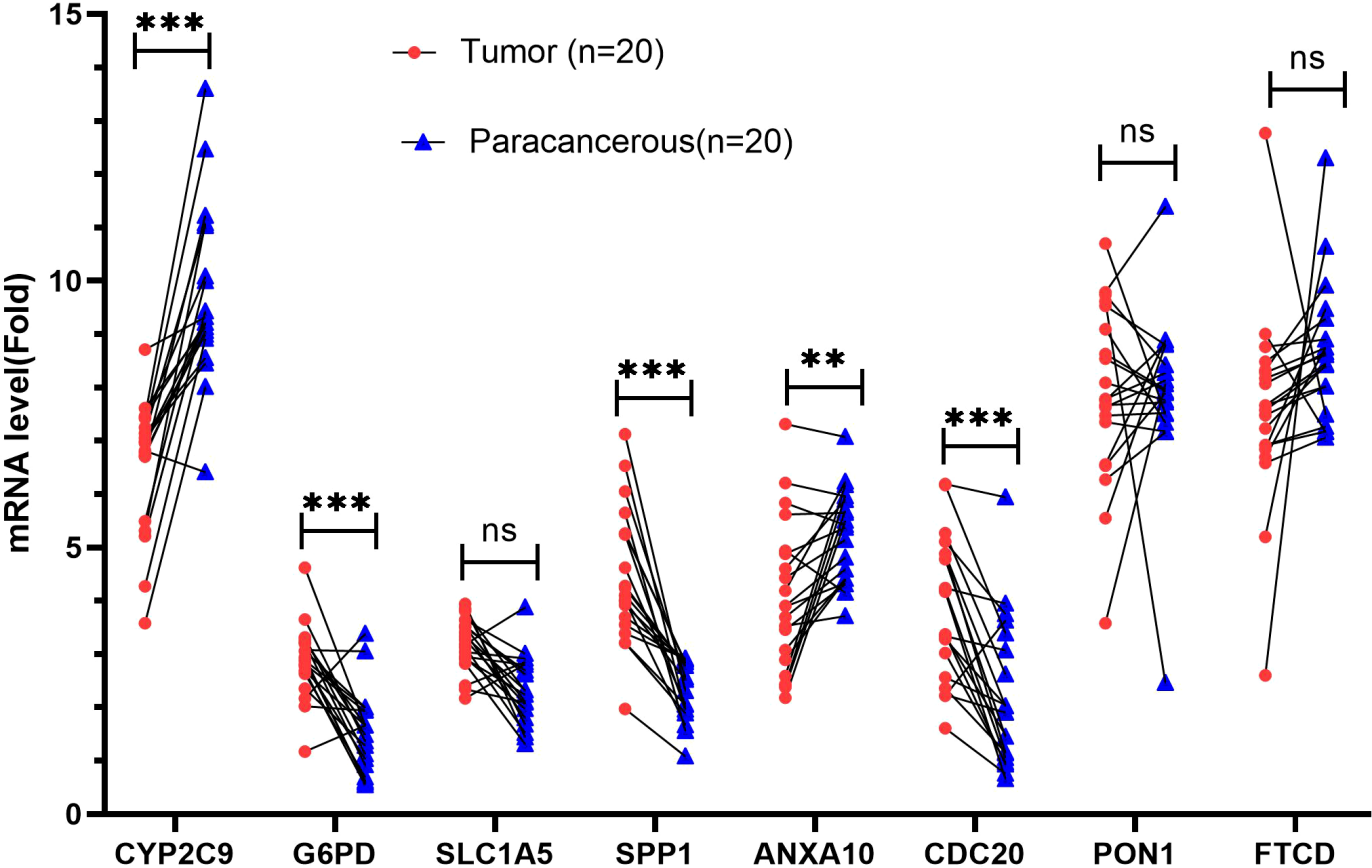
Validation of mRNA expression by real-time PCR. mRNA expression of eight genes associated with MARCH ligase in 20 HCC tissues and paracancerous tissues. ns, not statistically significant; **p< 0.01; ***p< 0.001.

To further verify the expression of risk genes in HCC, quantitative real-time polymerase chain reaction (qRT-PCR) was used to analyze the expression of MARCH ligase-related genes (*G6PD*, *SLC1A5*, *SPP1*, and *CDC20*). We verified the mRNA expression levels in 20 HCC and paracancerous tissues. The results showed that the expression of *G6PD*, *SPP1*, and *CDC20* in HCC tissues was higher than that in paracancerous tissues, while the expression of *CYP2C9* and *ANXA10* was lower than that in paracancerous tissues (**Figure 13**). The qRT-PCR results are mostly consistent with the group according to risk score.

## Discussion

Because of its high incidence rate and poor prognosis, HCC is regarded as one of the most malignant types of liver cancer (25). Therefore, finding predictive prognostic biomarkers for HCC to improve the clinical outcomes of HCC patients is of great significance. It has been reported that MARCH ligases play critical roles in tumor progression (26). Thus, a comprehensive analysis of the clinical significance, immune infiltration, and biological role of MARCH ligases can provide a new direction for the clinical treatment and research on HCC.

Through NMF algorithm clustering, we identified two MARCH ligase-related patterns. Compared with the C2 pattern, the C1 pattern indicated a poor OS or PFS probability. Moreover, the ratio of HCC patients with advanced T stages had higher levels in the C1 pattern. In addition, distinct immune cell infiltration and biological pathway enrichment were shown in these two MARCH ligase-related patterns. In the TCGA-HCC cohort, we found that the C1 pattern had more immune-related mutations and major MHC molecules. Recent studies have shown that the expression of HLA class I molecules in tumor cells is often associated with tumor escape from the immune system (27). A lack of HLA class I expression in tumor cells can have a negative impact on immunotherapy (28). These results indicate that MARCH ligase may affect the development of HCC by regulating the expression of MHC molecules in HCC. The evidence above certified that the MARCH ligase may play a significant role in regulating the immune landscape of HCC.

Next, we studied the DEGs between the C1 and C2 patterns. We established a prognostic signature (risk score) to evaluate and quantify HCC individuals, including eight MARCH ligase-related genes (*CYP2C9*, *G6PD*, *SLC1A5*, *SPP1*, *ANXA10*, *CDC20*, *PON1*, and *FTCD*) in the TCGA-HCC cohort. Based on the defined risk score, HCC patients were divided into high- and low-risk groups, and a series of analyses were performed. According to survival analysis, patients in the high-risk group had a worse OS than those in the low-risk group, suggesting that risk score is relevant to tumor progression or poor prognosis events. It was also proven in a separate external GEO-HCC cohort. Through the analysis of univariate Cox regression and multivariate Cox regression, we proved that risk score was an excellent independent prognostic indicator both in the TCGA-HCC cohort and in the GEO-HCC cohort. *CYP2C9* is a drug-metabolizing enzyme gene (DME gene) that regulates cell growth, apoptosis, differentiation, and homeostasis and is involved in hepatocarcinogenesis (29). Previous studies revealed that *G6PD* is a key energy metabolism gene for HCC and contributes to tumor proliferation, migration, and invasion (30, 31). *SLC1A5* is highly associated with the infiltration of tumor immune cells. Its expression can be a reliable prognostic biomarker in numerous cancers, especially in HCC (32). *SPP1* promotes the migration of HCC cells (33). *ANXA10* is related to the malignant phenotype of liver cells (34). *CDC20* assumes crucial functions of cells in the anaphase of mitosis (35), and its expression is upregulated in HCC (36). *PON1* not only can reduce the inhibition of leukocyte adhesion and chronic inflammation of vascular walls (especially macrophages and monocytes) but also participate in cell cholesterol synthesis (37) and result in tumor invasion/metastasis (38). The *FTCD* gene is downregulated in HCC tumor tissues. Therefore, as a reliable diagnostic biomarker, it distinguishes between early HCC and benign tumors (39).

To further verify the above results, qRT-PCR was conducted. The results showed that the expression of *G6PD*, *SPP1*, and *CDC20* in HCC tissues was increased compared with that in paracancerous tissues, while the expression of *CYP2C9* and *ANXA10* was decreased compared with that in paracancerous tissues. The results are mostly consistent with the group according to risk score. However, the *SLC1A5* and *FTCD* genes showed no significant difference. This lack of significance may be related to the small sample size and the expression of the protein that is not parallel to the mRNA.

It has been demonstrated that CD4^+^ T cells (40), CD8^+^ T cells (41), and NK cells (42) in HCC are protective factors. In addition, significantly reduced infiltration of CD4^+^ T cells, CD8^+^ T cells, and NK cells may trigger tumor immune evasion and ultimately lead to the progression, high recurrence, and poor prognosis of HCC based on their functions (43). Moreover, CD4^+^ T cells, CD8^+^ T cells, and NK cells are important cells in immunotherapy. CD4^+^ T cells are a key contributor to PD-L1/PD-1 blockade immunotherapy efficacy (44). Activated CD8^+^ cytotoxic T cells can release IFN-γ. Upon IFN-γ stimulation, PD-L1 is expressed on tumor cells (45). NK cells contribute to the effects of PD-1/PD-L1 blockade along with cytotoxic T cells (46). Interestingly, we found that patients in both the TCGA dataset and the GEO dataset with low-risk scores had lower CD4^+^ T-cell, CD8^+^ T-cell, and NK-cell infiltration levels than patients with high-risk scores in the immune microenvironment. Furthermore, the single-cell sequencing results showed that the low-risk group had higher T lymphocytes and B lymphocytes than the high-risk group. These results suggest that risk score may affect the prognosis of patients *via* the TIME.

Research has indicated that cancer patients are more likely to obtain long-term and effective responses from immunotherapy with high TMB (47, 48). In our research, the high-risk group had a higher TMB than the low-risk group, which we also confirmed in the TCGA dataset and the GEO dataset. However, compared with the high-risk group, patients in the low-risk group had more immune-related mutations. This is consistent with the results of immune infiltration mentioned above.

ICIs, chemotherapy, and TACE are effective clinical strategies for treating advanced HCC. In this study, we confirmed that the IC_50_ values of the AKT inhibitor VIII, gefitinib, and nilotinib were higher in the high-risk group than in the low-risk group, while the IC_50_ values of cisplatin and gemcitabine were lower in the high-risk group than in the low-risk group. The above results demonstrated that risk score is an important biomarker to assess immune status. Thus, we sought to further investigate the response rate to ICIs of risk score based on the TIDE score. The high-risk group had a lower TIDE score, which suggested that the high-risk group may have a higher response to ICI treatment. The findings above suggest that patients in the low-risk group may benefit more from the therapeutic regimens of TACE. Taken together, we believe that risk score may have important guiding significance for HCC treatment, including chemotherapy, immunotherapy, and TACE.

In addition, we performed GSVA and confirmed the result that the low-risk group was related to metabolism, and the high-risk group was relevant to carcinogenic activation-related signaling pathways, such as DNA repair, mTORC1 signaling, and UPR. Interestingly, all of these signaling pathways contribute to the progression of HCC. DNA repair (49), mTORC1 signaling (50), and UPR (51) are associated with unique characteristics and worse survival in HCC patients. DNA repair, a mechanism that allows cells to live longer, can lead to the accumulation of genetic lesions that can lead to the formation of cancer (52). Aberrant activation of MTORC1 signaling *via* enhanced cell survival and metastasis results in tumorigenesis and cancer progression (53). The UPR signaling pathway has been recognized to promote tumor cell proliferation by limiting oxidation DNA damage (54), and the UPR promotes HCC immune escape by transferring specific miRNAs to infiltrated macrophages in the tumor microenvironment (55). In addition, according to the GeneCards database (https://www.genecards.org/), MARCH E3 ligases are mainly located in the ER and mitochondria. Therefore, it was suggested that the expression of MARCH E3 ligases may be regulated by various tumor-related stresses. The results in this work point to future directions of the study.

Collectively, by comprehensively evaluating the molecular, cellular, and clinical characteristics of HCC patients, risk score can quantify and individualize the phenotypes of HCC patients. The risk score may have important implications for the selection of ICIs, chemotherapy, and TACE strategies for HCC patients. However, our study still has limitations that need to be further refined. As a retrospective study, multicenter, large-sample sizes, and prospective studies are required to confirm our results further. Moreover, further experimental studies are needed to refine our understanding of the relationship between MARCH ligases and HCC.

## Data availability statement

The datasets presented in this study can be found in online repositories. The names of the repository/repositories and accession number(s) can be found in the article/**Supplementary Material**.

## Ethics statement

This study was reviewed and approved by the Ethics Committee of the Subei People’s Hospital of Jiangsu Province, Clinical Medical College, Yangzhou University. The patients/participants provided their written informed consent to participate in this study.

## Author contributions

JC, DT, JZ, DB, and CZ designed and implemented the research. GJ, SJ, BS, HT, YT, AW, QW, and RL collated and analyzed the data. JC, DT, JZ, DB, and CZ provided technical support. CZ and JZ provided the language polishing for this article. DT wrote the manuscript. DB and JC revised the manuscript. All authors contributed to the article and approved the submitted version.

## Funding

This study was supported by The National Natural Science Foundation of China (grant number 81871909); “13th five-year Plan” Science and Education strong Health Project Innovation team of Yangzhou (YZCXTD201801); Provincial-level discipline leader of the NJPH (DTRC201809); Cross-cooperation special projects of the NJPH(YJCHZ-2021-08); Postgraduate Research and Practice Innovation Program of Jiangsu Province (KYCX22_3568).

## Acknowledgments

We thank The Cancer Genome Atlas (TCGA) and Gene Expression Omnibus (GEO) for data support, gene set enrichment analysis (GSEA), and gene ontology (GO) for functional enrichment.

## Conflict of interest

The authors declare that the research was conducted in the absence of any commercial or financial relationships that could be construed as a potential conflict of interest.

## Publisher’s note

All claims expressed in this article are solely those of the authors and do not necessarily represent those of their affiliated organizations, or those of the publisher, the editors and the reviewers. Any product that may be evaluated in this article, or claim that may be made by its manufacturer, is not guaranteed or endorsed by the publisher.

## Supplementary material

Supplementary Figure 1 Flow chart of our study.

Supplementary Figure 2 mRNA expression of MARCH ligases in 40 HCC tissues and paracancerous tissues in the TCGA-HCC cohort. ns not statistically significant, ** p < 0.01, *** p < 0.001.

Supplementary Figure 3
**(A)** Heatmap of DEGs between the C1 and C2 patterns. **(B)** Functional enrichment analyses of DEGs between C1 and C2 patterns.

Supplementary Figure 4
**(A)** Gene expression levels in each cell of the 4 samples were in the range of 50-9000, and the distribution was relatively uniform. At the same time, we found that the percentage of mitochondrial genes was almost 0. **(B)** Cells are evenly distributed in the four samples, and the number of genes is positively correlated with the expression level of genes, with a correlation of 0.8. **(C)** We selected 300 hypervariable genes from all the genes, which are in red, and the first 10 genes were flagged. **(D)** After dimension reduction through PCA, we found that the cells were clustered into 12 clusters.
