# Supplementary figures and images for: Comprehensive analysis of the clinical significance, immune infiltration, and biological role of MARCH ligases in HCC
Source: Front Immunol. 2022 Oct 3;13:997265. doi: 10.3389/fimmu.2022.997265 (PMC9573977; doi:10.3389/fimmu.2022.997265)

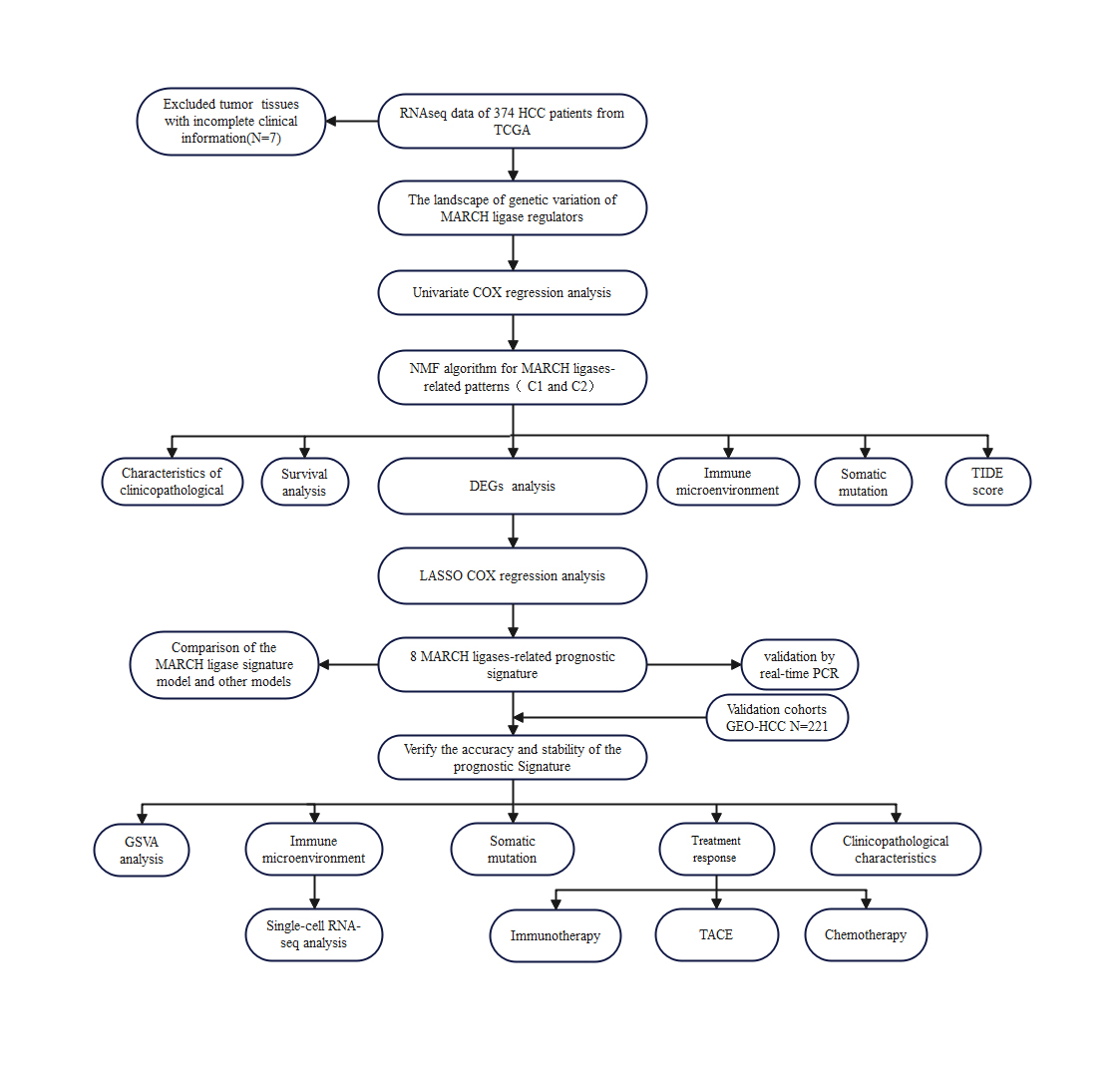

Supplement: Supplementary Figure 1 — Flow chart of our study. [file Image_1.jpeg]

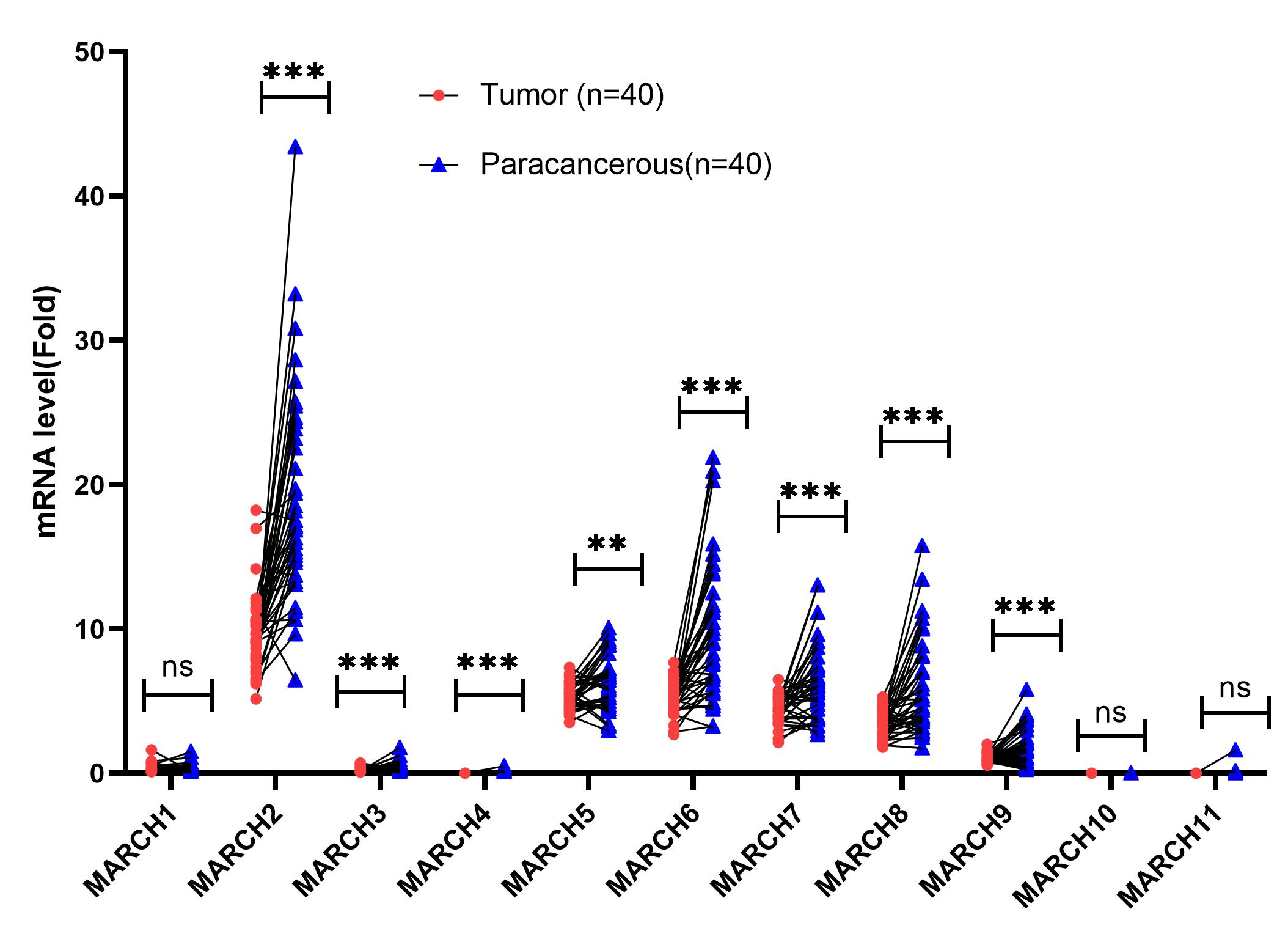

Supplement: Supplementary Figure 2 — mRNA expression of MARCH ligases in 40 HCC tissues and paracancerous tissues in the TCGA-HCC cohort. ns not statistically significant, ** p < 0.01, *** p < 0.001. [file Image_2.jpeg]

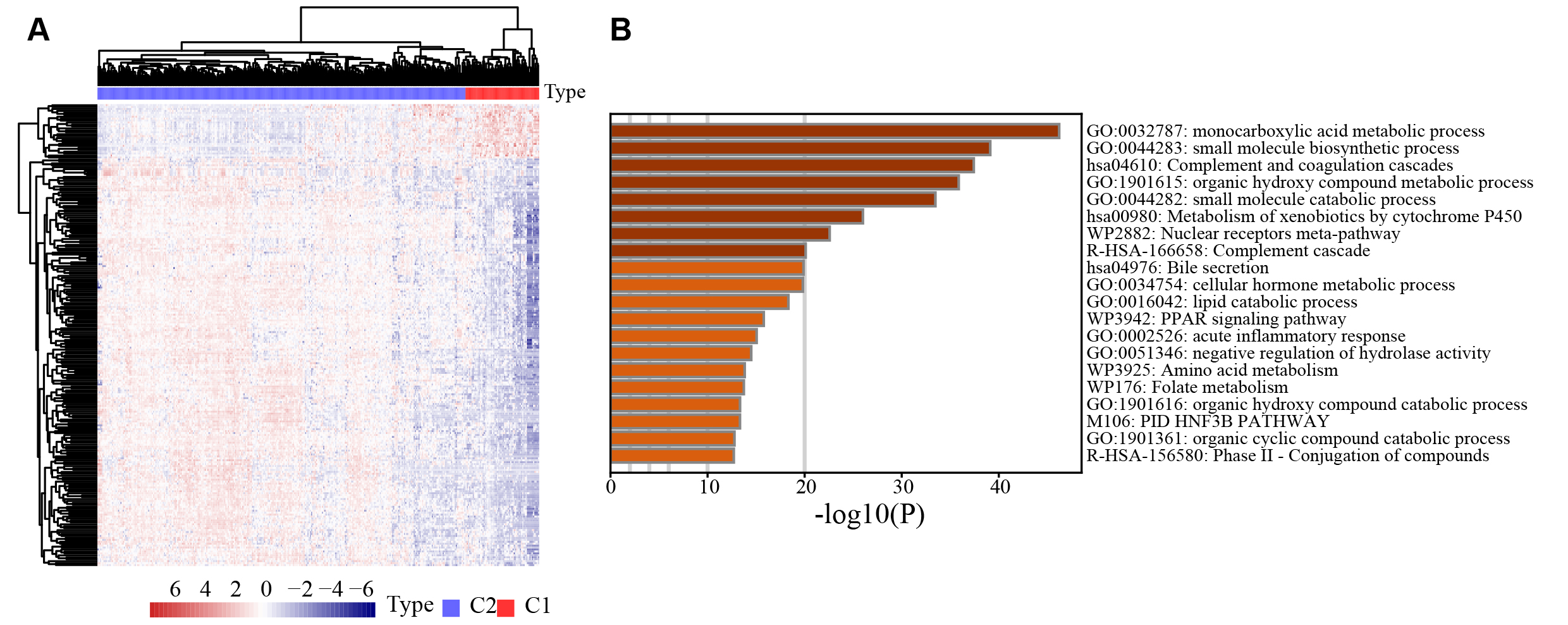

Supplement: Supplementary Figure 3 — (A) Heatmap of DEGs between the C1 and C2 patterns. (B) Functional enrichment analyses of DEGs between C1 and C2 patterns. [file Image_3.jpeg]

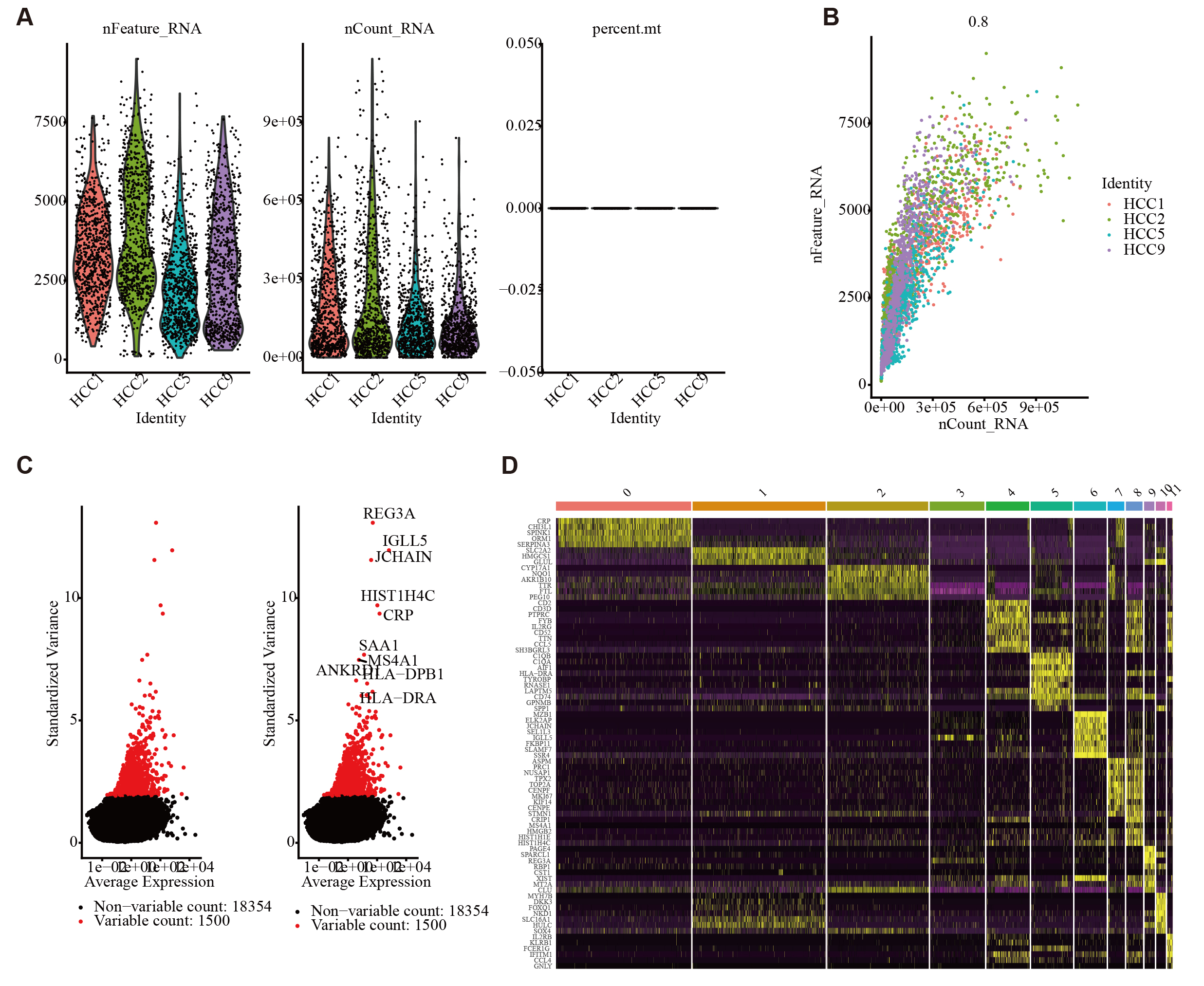

Supplement: Supplementary Figure 4 — (A) Gene expression levels in each cell of the 4 samples were in the range of 50-9000, and the distribution was relatively uniform. At the same time, we found that the percentage of mitochondrial genes was almost 0. (B) Cells are evenly distributed in the four samples, and the number of genes is positively correlated with the expression level of genes, with a correlation of 0.8. (C) We selected 300 hypervariable genes from all the genes, which are in red, and the first 10 genes were flagged. (D) After dimension reduction through PCA, we found that the cells were clustered into 12 clusters. [file Image_4.jpeg]
