# Supplementary material for: Comprehensive analysis of the clinical significance, immune infiltration, and biological role of MARCH ligases in HCC
Source: Front Immunol. 2022 Oct 3;13:997265. doi: 10.3389/fimmu.2022.997265 (PMC9573977; doi:10.3389/fimmu.2022.997265)
Supplement: Supplementary file 5 [file Table_1.docx]

**Supplementary Table 1** List of the specific primers for 8 prognostic Gene

| Gene | Forward | Reverse |
| --- | --- | --- |
| CYP2C9 | 5′- TGGATGAAGGTGGCAATTTT -3′ | 5’- GGGCTTCTCCCA CACAAAT -3’ |
| G6PD | 5’- CGAGGCCGTCACCAAGAAC -3’ | 5’- GTAGTGGTCGATGCGGTAGA -3’ |
| SLC1A5 | 5’- CAACCTGGTGTCAGCAGCCTT -3’ | 5’- GCACCGTCCATGTTGACGGTG -3’ |
| SPP1 | 5’- CGAGGTGATAGTGTGGTTTATGG -3’ | 5’- GCACCATTCAACTCCTCGCTTTC -3’ |
| ANXA10 | 5’- GTCCTATGGGAAGCCTGTCA -3’ | 5’- GCACCATTCAACTCCTCGCTTTC -3’ |
| CDC20 | 5’- AACGGCAGGACUCCGGGCCGA -3’ | 5’- AAUGGCCAGUGGUGGUAAUGA -3’ |
| PON1 | 5’- GGATCCATGGCGAAGCTGATTGCGTCTAC -3’ | 5’- GCACCATTCAACTCCTCGCTTTC -3’ |
| FTCD | 5’- GCGTGTTTGGCGCATATT -3’ | 5’- GATGGTGGATCTGGTCCTTAAA -3’ |
